# Answer to the Photo Quiz: Unexpected finding in a Gram stain

**DOI:** 10.1128/jcm.00642-23

**Published:** 2023-11-21

**Authors:** Cristina Bayo Sánchez, Iosu Razquin Olazarán, Carmen Martín Salas, María Eugenia Portillo Bordonabe, Carmen Ezpeleta Baquedano

**Affiliations:** 1 Servicio de Microbiología, Hospital Universitario de Navarra, Pamplona, Navarra, Spain; Mayo Clinic Minnesota, Rochester, Minnesota, USA

**Keywords:** *Strongyloides*, disseminated infection, Gram stain

## ANSWER TO PHOTO QUIZ

The definitive diagnosis was disseminated strongyloidiasis with concurrent *Escherichia coli* bacteremia. Larvae of *Strongyloides stercoralis* were also observed in stool smears after a centrifugation concentration method. This diagnosis was confirmed by qPCR detection of *Strongyloides* spp. using the Allplex GI-Helminth (I) Assay (Seegene) (1) in the tracheal aspirate, stool, ascitic fluid, and cutaneous biopsies. Despite appropriate treatment, the patient died after 10 days of hospitalization in the intensive care unit.

*S. stercoralis* is a soil-transmitted nematode that is endemic in tropical and subtropical countries, especially in areas with inadequate sanitary conditions, and it is recognized as a neglected tropical disease (2). Our case is a disseminated infection probably acquired in Ecuador, so we can assume that it is caused by *S. stercoralis*. Other species that infect humans do not produce autoinfection or dissemination and are also distributed in countries other than the Americas (3). Strongyloidiasis has been estimated to affect over 600 million people worldwide, although is less prevalent in developed countries (4). In Spain, nearly 97% of the cases described had been acquired in the Valencia region, mainly in farm workers (5).

*Strongyloides* infection occurs when soil-dwelling filariform larvae penetrate the skin, especially through barefoot contact. They pass into the venous circulation and migrate to the heart and lungs. Then, they are expectorated into sputum and swallowed into the small intestine, or randomly migrate there without lung involvement. Here they mature into adult females and reproduce by parthenogenesis. Finally, rhabditiform larvae, which hatch from eggs within the intestine, are excreted in stools. A small number will develop into autoinfective filariform larvae (L3a) in the gut, resulting in autoinfection. This unique property allows infection to persist for decades (chronic strongyloidiasis) and cause overwhelming infection if people become immunosuppressed later in life, leading to *Strongyloides* Hyperinfection Syndrome (SHS) or disseminated infection (6, 7). Concurrent bacterial infections, mostly from Enterobacterales, often occur in SHS and disseminated infection, with poor outcomes and a high mortality rate, as in our patient’s case. The absence of eosinophilia cannot rule out strongyloidiasis, especially in immunocompromised individuals. Immunosuppression due to corticosteroids is the leading cause of severe strongyloidiasis (2), so asymptomatic *Strongyloides* infection in patients treated with dexamethasone needs to be considered to avoid precipitating SHS and disseminated strongyloidiasis (7).
